# Supplementary material for: Reversal of cancer gene expression correlates with drug efficacy and reveals therapeutic targets
Source: Nat Commun. 2017 Jul 12;8:16022. doi: 10.1038/ncomms16022 (PMC5510182; doi:10.1038/ncomms16022)
Supplement: Supplementary Information [file ncomms16022-s1.pdf]

Type of file: PDF
Size of file: 0 KB
Title of file for HTML: Supplementary Information
Description: Supplementary figures, supplementary table and supplementary methods.

Type of file: XLSX
Size of file: 0 KB
Title of file for HTML: Supplementary Data 1
Description: Cell line mappings between CCLE, LINCS, ChEMBL, and CTRP in BRCA, LIHC, COAD, and ER+ BRCA.

Type of file: XLSX
Size of file: 0 KB
Title of file for HTML: Supplementary Data 2
Description: Disease gene expression signatures for BCRA, COAD, and LIHC. Log 2 fold change > 1.5 and adjusted p value < 0.001 were used to identify significantly differentially expressed genes between tumors and adjacent normal tissues.

Type of file: XLSX
Size of file: 0 KB
Title of file for HTML: Supplementary Data 3
Description: RGES and IC50 in three cancer cell lines. The data was used to generate the plots in Fig. 3.

Type of file: XLSX
Size of file: 0 KB
Title of file for HTML: Supplementary Data 4
Description: Performance of different methods used to compute RGES and to summarize RGES.

Type of file: XLSX
Size of file: 0 KB
Title of file for HTML: Supplementary Data 5
Description: sRGES and IC_{50} in three cancers. The data was used to generate the plots in Fig. 4.

Type of file: XLSX
Size of file: 0 KB
Title of file for HTML: Supplementary Data 6
Description: Drug predictions for LIHC. The drug hits selected for validation were highlighted.

Type of file: XLSX
Size of file: 0 KB
Title of file for HTML: Supplementary Data 7
Description: Genes reversed by effective compounds.

Type of file: XLSX
Size of file: 0 KB
Title of file for HTML: Supplementary Data 8
Description: Drug IC_{50} in cancer cell lines.

Type of file: PDF
Size of file: 0 KB

Title of file for HTML: Peer review file
Description:

Supplementary Methods

Data harmonization

We presented an informatics pipeline to integrate patient samples, cancer cell lines, and compounds from four public databases TCGA, LINCS, ChEMBL and CCLE. Supplementary Fig. 9 illustrates primary entities and their connections across databases. TCGA includes tissue sample gene expression profiles, LINCS includes perturbagen-mediated gene expression profiles, ChEMBL includes drug activity in cancer cells, and CCLE includes cancer cell gene expression profiles. Each cell line in CCLE, if possible, was first manually assigned a cancer type listed in TCGA based on its primary name, primary site, histology, and histology subtype. Cell lines in CCLE, ChEMBL and LINCS were mapped using their name followed by manual inspection. Compounds in LINCS and ChEMBL were mapped using InChi-keys.

Disease selection

We first chose the cancers that have at least ten tumors and ten adjacent normal tissues from TCGA, resulting in fourteen cancers left for computing disease signatures (Bladder Urothelial Carcinoma-BLCA, Breast invasive carcinoma-BRCA, Colon adenocarcinoma-COAD, Head and Neck squamous cell carcinoma-HNSC, Kidney Chromophobe-KICH, Kidney renal clear cell carcinoma-KIRC, Kidney renal papillary cell carcinoma-KIRP, Liver hepatocellular carcinoma-LIHC, Lung adenocarcinoma-LUAD, Lung squamous cell carcinoma-LUSC, Prostate adenocarcinoma-PRAD, Stomach adenocarcinoma-STAD, Thyroid carcinoma-THCA, Uterine Corpus Endometrial Carcinoma-UCEC). These signatures were mapped to the LINCS landmark genes. We further manually

matched cancers to their cancer cell lines based on cell line characteristics provided in CCLE. We found ten cancers (BLCA, BRCA, COAD, KIRC, LIHC, LUAD, LUSC, PRAD, STAD, THCA) that matched to at least one cell line of its lineage. We then retrieved drug efficacy data for these cell lines from ChEMBL. As a result, only BRCA, LIHC and COAD had > 50 differentially expressed genes and > 30 compounds with at least one expression profile and at least one IC_{50} in cancer cell lines.

We selected diseases with over 30 compounds in order to get a sufficient statistical power in the following analysis. We selected diseases with over 50 differentially expressed genes based on our empirical experience of using CMap data and preliminary evaluation. In our evaluation, we ranked disease genes based on fold change and selected a certain number of genes on each side (up/down) to build a disease signature. For each signature, we measured the correlation between RGES and IC_{50} in individual cancer cell lines (BRCA: MCF7, COAD: HT29, LIHC: HepG2). We observed that as the size of gene set we chose was increasing, the correlation increased and then converged (Supplementary Fig. 10). In the three cancers, when the size of the gene set of one side was about 25, the correlation did not increase. Therefore, we only chose the diseases with > 50 differentially expressed genes. We note that we did not take into account other factors (e.g., fold change, q-value), and the threshold of choosing the number of differentially expressed genes could be optimized.

Correlation between tumor samples and cell lines

As gene expression in tumor samples from TCGA and in cell lines from CCLE was profiled using two different technologies and processed using different methods, we used the ranked-based Spearman correlation to assess the similarity in gene expression between cell lines and tumor samples. The top 5000 genes ranked by interquartile range across all cell lines were used. For each cell line, the median of its correlations with all tumor samples was considered. For each tumor, the median of its correlations with all cancer cell lines with the same lineage was considered. Among these samples, 2.4% (BRCA), 2.8% (LIHC), and 0.9% (COAD) are not correlated with cancer cell lines (adjusted P value < 0.05).

Method to summarize RGES

We used the following formula to summarize the RGES of each drug:

$$sRGES = \sum_{i}^{N} (RGES(i) + f(dose(i), time(i))) \times w(i) \Big/ N$$

First, we considered the effect of drug concentration and treatment duration on RGES. The condition with 10 μM and 24 h treatment is set as a reference condition and all other conditions are set as target conditions. We assume that the difference in RGES of one drug between a target condition and a reference condition in one cell line is mainly dependent on its concentration and treatment duration. In order to estimate the difference, we used the drugs, which were profiled in the same cell line with at least one target condition and at least one reference condition. Suppose profile P_i was obtained from a target condition, and profile P_j was obtained from a reference condition. Profile P_i was

paired up with profile P_j if both share the same drug and cell line. We used a simple awarding function to infer RGES $f(dose(i), time(i))$

$$f(dose(i), time(i)) = \begin{cases} \alpha,\ dose(i) < 10um\ and\ time(i) < 24h \\ \beta,\ dose(i) < 10um\ and\ time(i) \geq 24h \\ \gamma, dose(i) \geq 10um\ and\ time(i) < 24h \\ 0, dose(i) \geq 10um\ and\ time(i) \geq 24h \end{cases}$$

Any profile from other conditions would receive awarding points, estimated by averaging the difference in RGES between the target group and reference group.

We next considered the effect of cell line on RGES. We weighted cell lines based on their relevance to tumor samples under study. Since the expression of some rare cell lines and normal cell lines was not profiled in CCLE, we excluded these cell lines (12 cell lines in total).

Comparison of the summarization method with existing methods

To indicate how well a particular perturbagen is connected to the query in a given number of cell lines at the LINCS cloud (query tool at http://apps.lincscloud.org), different score metrics (e.g., mean_rankpt_2, rankpt_1) were developed to summarize connectivity scores. The scores ranging from 100 (complete connection) to -100 (complete anti-connection) can be used to rank compounds. The query tool also gives the connectivity score of an individual drug profile for a given disease signature. We also examined if their summarized scores are correlated to drug efficacy. In addition, we used our method to summarize connectivity scores, and compared our method with those in LINCS.

We used the signatures of BRCA, LIHC, and COAD to query LINCS. The summary table and score table were downloaded from their website. We found that the connectivity scores from a few score metrics are positively correlated to IC_{50} (Supplementary Fig. 13). Among these, score_best4, which is the mean connectivity score across the four cell lines in which the perturbagen connected most strongly to the disease signature, gives the best correlation. Our observation is consistent with recommendations provided in the LINCS knowledge base. When our method was used to summarize the scores, the correlation increased dramatically 37% (BRCA: 0.30 to 0.41), 6% (COAD: 0.35 to 0.37), and 12% (LIHC: 0.49 to 0.55) (Supplementary Fig. 13). Our results suggest that our summarization method, which takes into account confounding factors (cell line and treatment conditions) outperforms the LINCS metrics, which do not take into account these factors.

Examination of vinblastine in BRCA

We downloaded the series matrix in GSE69845, where MCF7 was treated with vinblastine for multiple times. Each probe was annotated using Entrez Gene ID and quantile normalization was performed. Expression of multiple probes for the same gene was averaged. Samples annotated as DMSO were considered as the control group, and samples annotated as vinblastine as the treatment group. The mean difference between two groups was used to generate the drug signature, which was then compared with the whole genome signature of BRCA signature (without mapping to the LINCS landmark genes) to compute RGES.

Protein extraction and immunoblotting

Total protein from LIHC cell lines and animal tissues were extracted with the T-PER Tissue Protein Extraction Reagent supplemented with protease inhibitor (all from Thermo Fisher Scientific Inc., Rockford, IL). After centrifugation at 13,000 rpm for 15 minutes at 4°C, supernatants were collected and total protein concentration measured by BCA Protein Assay Kit (Pierce, Rockford, IL). The total protein lysates (10 µg for LIHC cells and 20 µg for animal tissues) were mixed with loading buffer, resolved on sodium dodecyl sulfate-polyacrylamide gels, and electrotransferred onto nitrocellulose membranes. The membranes were blocked for 1 hour at room temperature in 5% nonfat dry milk in Tris-buffered saline per 0.1% Tween, and then incubated with desired primary antibodies: anti-Survivin (NB500-201) from Novus Biological (Littleton, CO), anti-LRP6 (2560); anti-Axin1 (2074); anti-Axin2 (5863); anti-Cyclin-D1; anti-p21; anti-Bcl-2 (2870); anti-Bcl-xl (2764); anti-pSTAT3 (9145) from Cell Signaling (Danvers, MA), anti-GAPDH (SC-365062) from Santa Cruz Biotechnology (Santa Cruz, CA) overnight at 4°C. The appropriate horseradish peroxidase-conjugated secondary antibodies were then added and allowed to incubate for 2 hours at room temperature. The immunoreactive complexes were detected by using the Super-Signal West Pico Chemiluminescent or West Femto Maximum Sensitivity substrate (Thermo Fisher Scientific Inc., Rockford, IL), according to the manufacturer's protocols.

TOPflash luciferase reporter assay

The Huh7 and HepG2 cells were plated in 24-well plates, at a density of 2×10^5 cells in 500 µL media per well. The co-transfection solution (100 µL per well) was made by

mixing 750 ng of TOPflash reporter plasmid or 750 ng of FOPflash reporter plasmid with 50 ng TK-Renilla plasmid (for a total of 800 ng) and 2 μL of Lipofectamine 2000 Transfection Reagent (Invitrogen, Carlsbad, CA). The transfection mixture was incubated at room temperature for 15 minutes. Meanwhile, the growth media were removed from each well and 400 μL of serum-free DMEM was added, followed by 100 μL of the transfection mixture. After 6 hours of incubation, media plus transfection mixture were removed and replaced with fresh serum-free media containing pyrvinium pamoate at a final concentration of 0.8, 1.6, or 3.2 μM supplemented with 100 ng per mL of rhWNT3a ligand (R&D Systems, Minneapolis, MN). DMSO was used as the vehicle control. At 24 hours post-drug treatment, the cells were lysed in Passive Lysis Buffer (Promega, Madison, WI) and luciferase activities were measured using the Dual-Luciferase Assay System (Promega, Madison, WI) and a Veritas Microplate Luminometer (Turner Biosystems, Promega, Madison, WI). Firefly luciferase activity was normalized Renilla Luciferase activity. Control conditions were set to one, and fold activities are shown relative to this. All experimental conditions were assessed in triplicates, and the experiments were repeated at least three times.

Supplementary Table

Supplementary Table 1: *In vitro* efficacy data of four drug candidates predicted for LIHC in five LIHC cell lines.

	HepG2	Huh7	Hep3B	PLC5	Hep40	Median IC_{50} (μM)
strophanthidin	11.59	0.72	0.16	1.61	0.19	0.72
FCCP	7.84	2.67	0.29	1.78	0.87	1.78
CGK 733	6.12	2.96	3.18	9.51	2.73	3.18
pyrvinium pamoate	0.14	0.02	0.01	0.15	0.07	0.07

Supplementary Figures

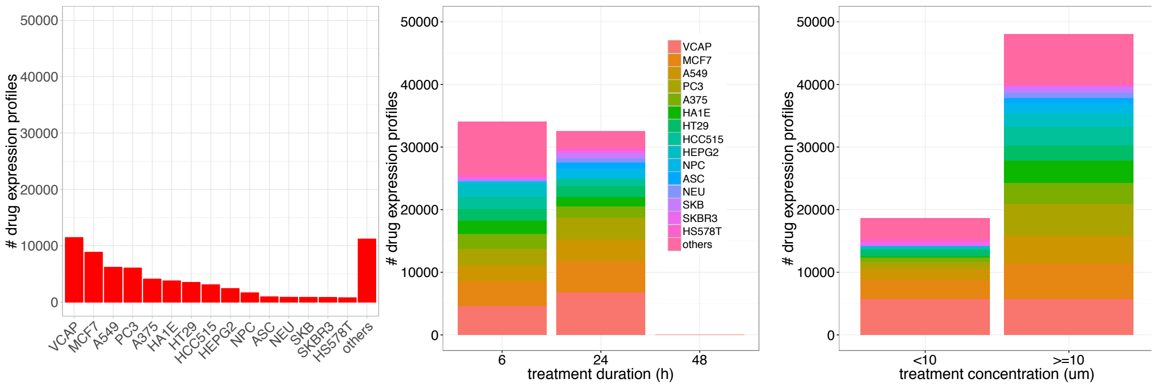

Supplementary Figure 1: Drug profile distribution across cell lines, treatment durations, and drug concentrations.

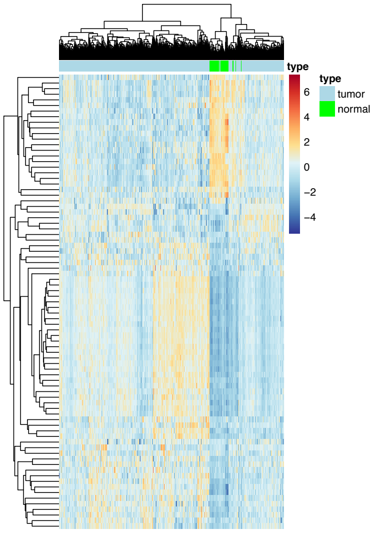

BRCA

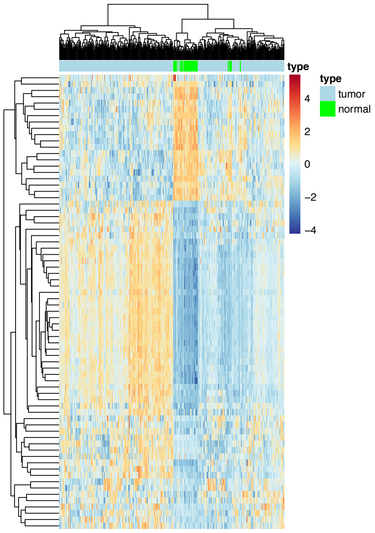

LIHC

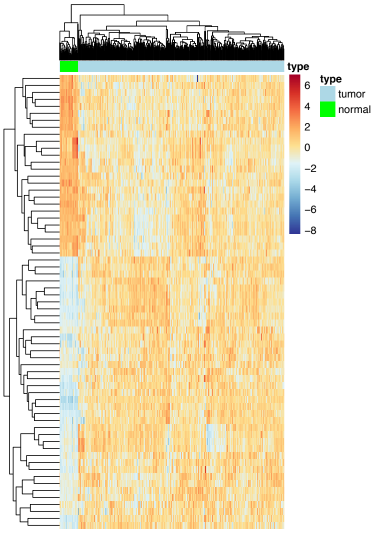

COAD

Supplementary Figure 2: Classify tumors and non-tumors using reduced disease signatures of BRCA, LIHC, and COAD.

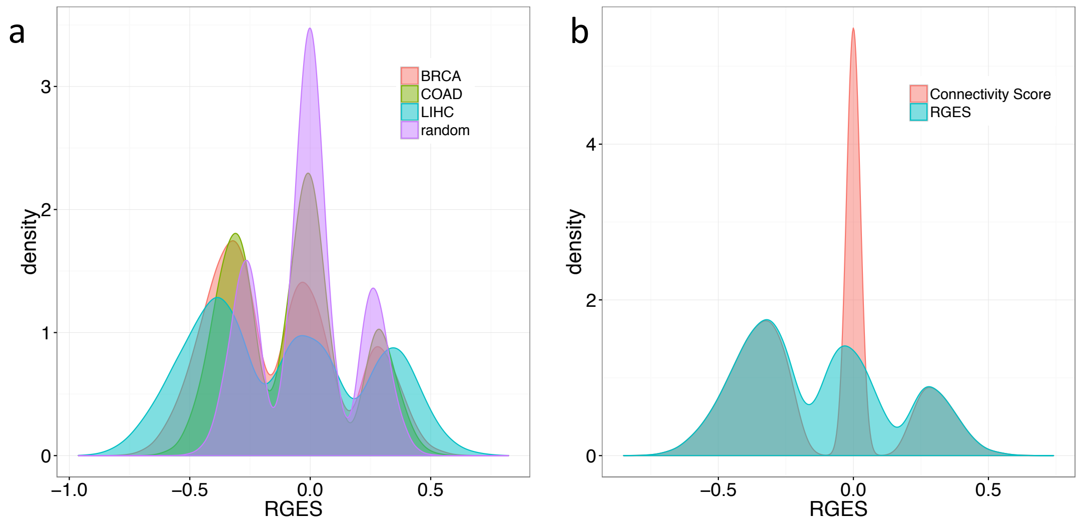

Supplementary Figure 3: (a) RGES distribution. Random represents the distribution from a signature composed of 70 genes randomly selected from the landmark genes. (b) RGES and connectivity score in BRCA predictions. Red represents the connectivity score distribution using the reported CMap method, and blue represents the RGES distribution adapted from the CMap method. It shows that a large number of connectivity scores are enriched at 0.

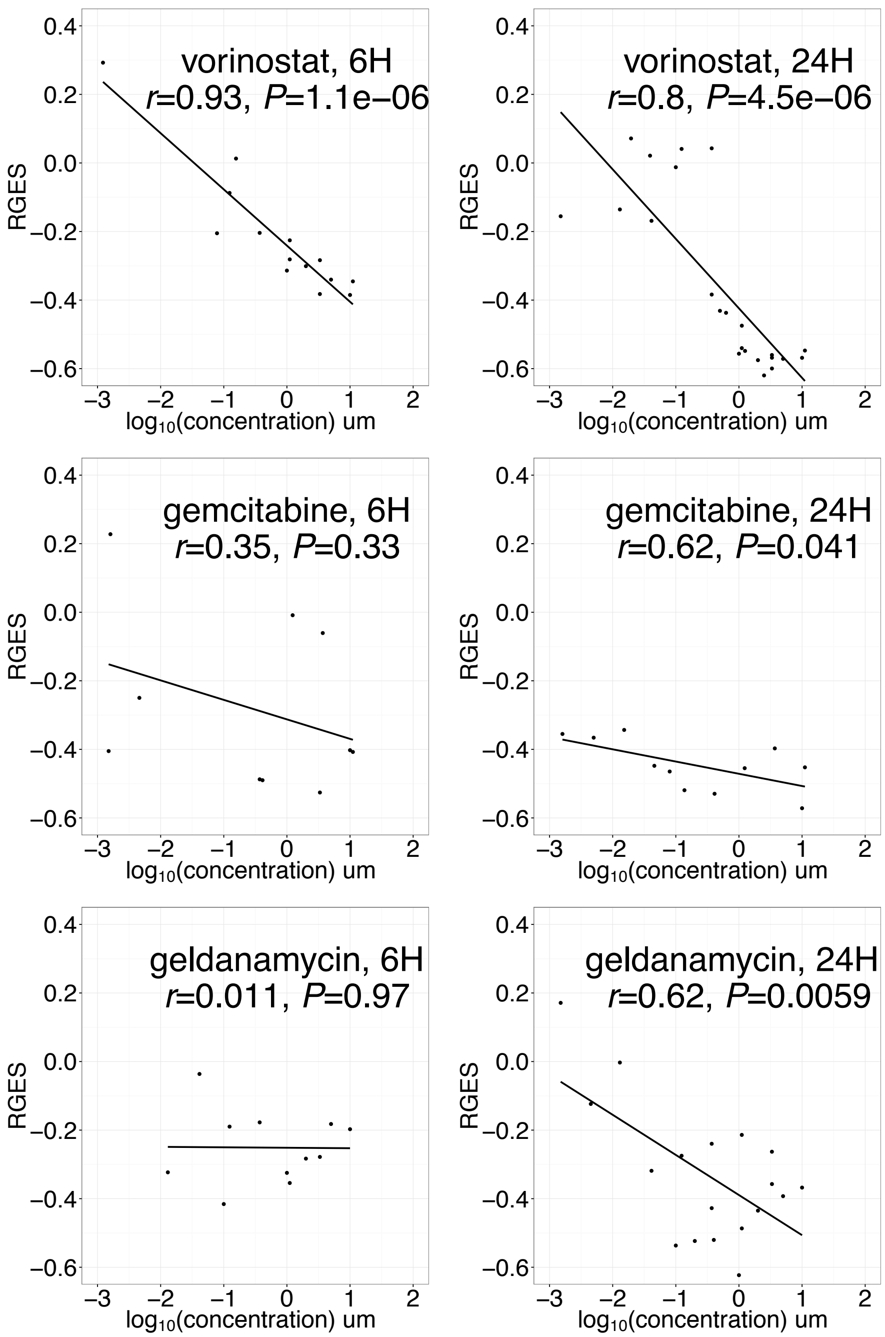

Supplementary Figure 4: Correlation between RGES and drug concentration under different treatment durations. RGES was computed using the BRCA disease gene expression signature and drug expression profiles in MCF7 cell line.

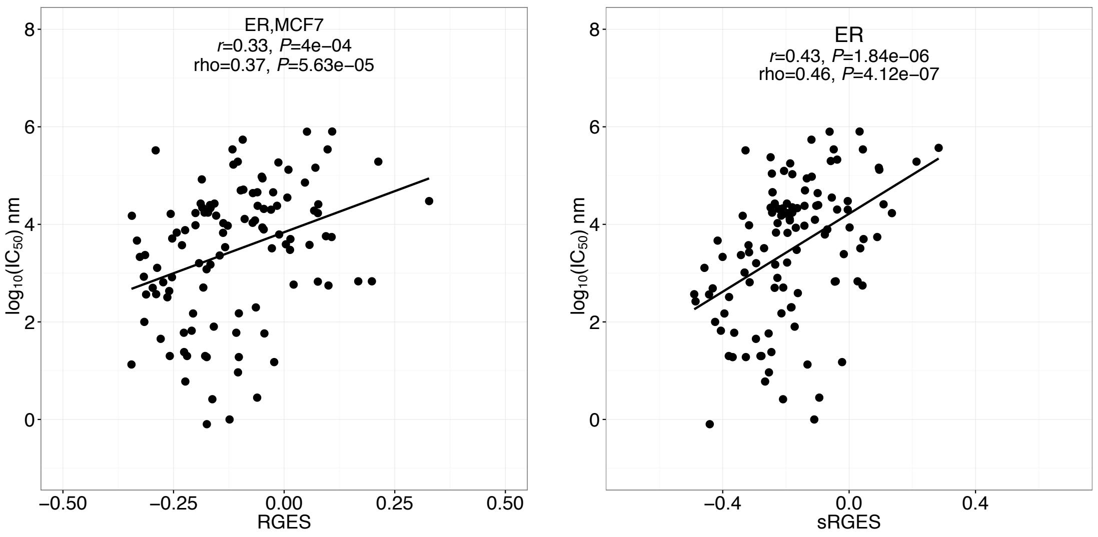

Supplementary Figure 5: Correlation between drug efficacy and (a) RGES in ER positive BRCA in MCF7 cells, and (b) sRGES in ER positive BRCA. Median IC_{50} was used when one compound has multiple IC_{50s} from different studies. ANOVA and Spearman correlation were used to measure correlation between RGES and drug efficacy.

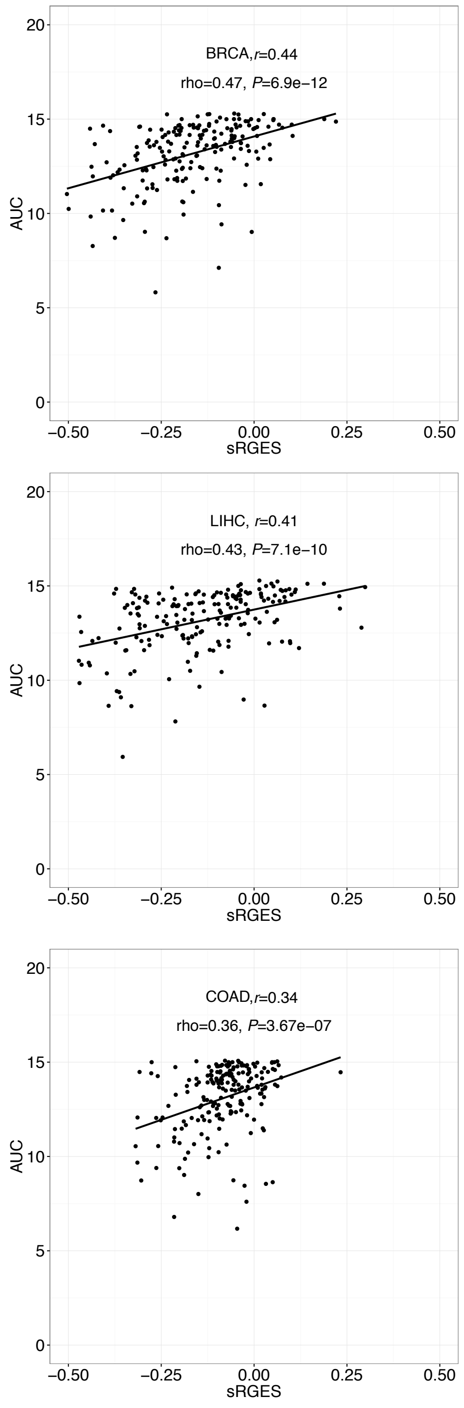

Supplementary Figure 6: Correlation between AUC and sRGES. AUC data were retrieved from CTRP. Median was used to summarize AUC across multiple cell lines. ANOVA and Spearman correlation were used to measure correlation between sRGES and drug efficacy.

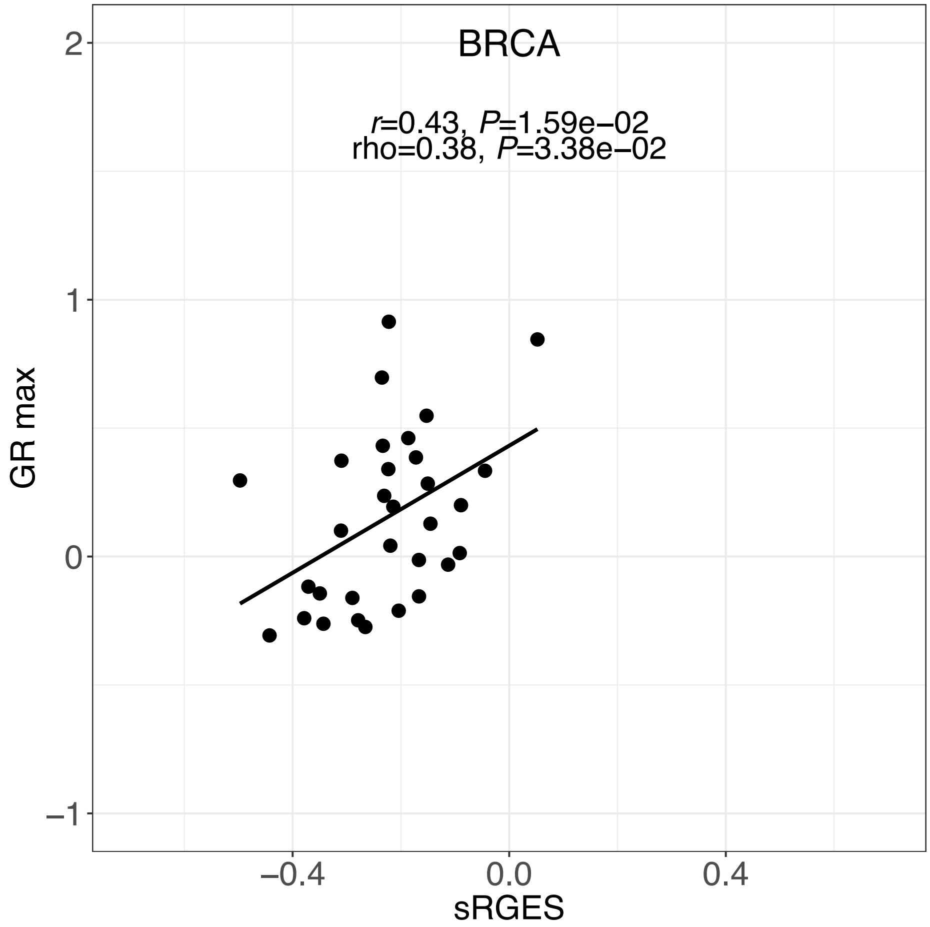

Supplementary Figure 7: Correlation between GR max and sRGES. GR max data were retrieved from the LINCS Pilot Phase Joint Project (http://www.grcalculator.org/grbrowser/), which only includes breast cancer cell lines. Median was used to summarize GR max across multiple cell lines. ANOVA and Spearman correlation were used to measure correlation between sRGES and drug efficacy.

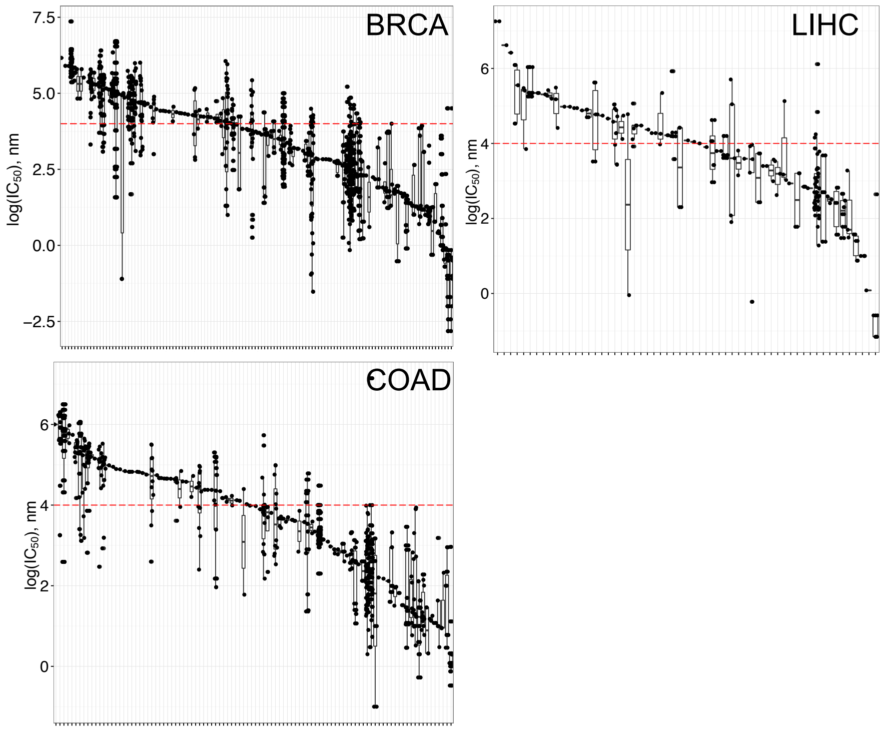

Supplementary Figure 8: IC_{50} distribution in BRCA, LIHC, and COAD cell lines. Each boxplot represents one compound. The IC_{50}s of one compound vary across different cell lines or studies.

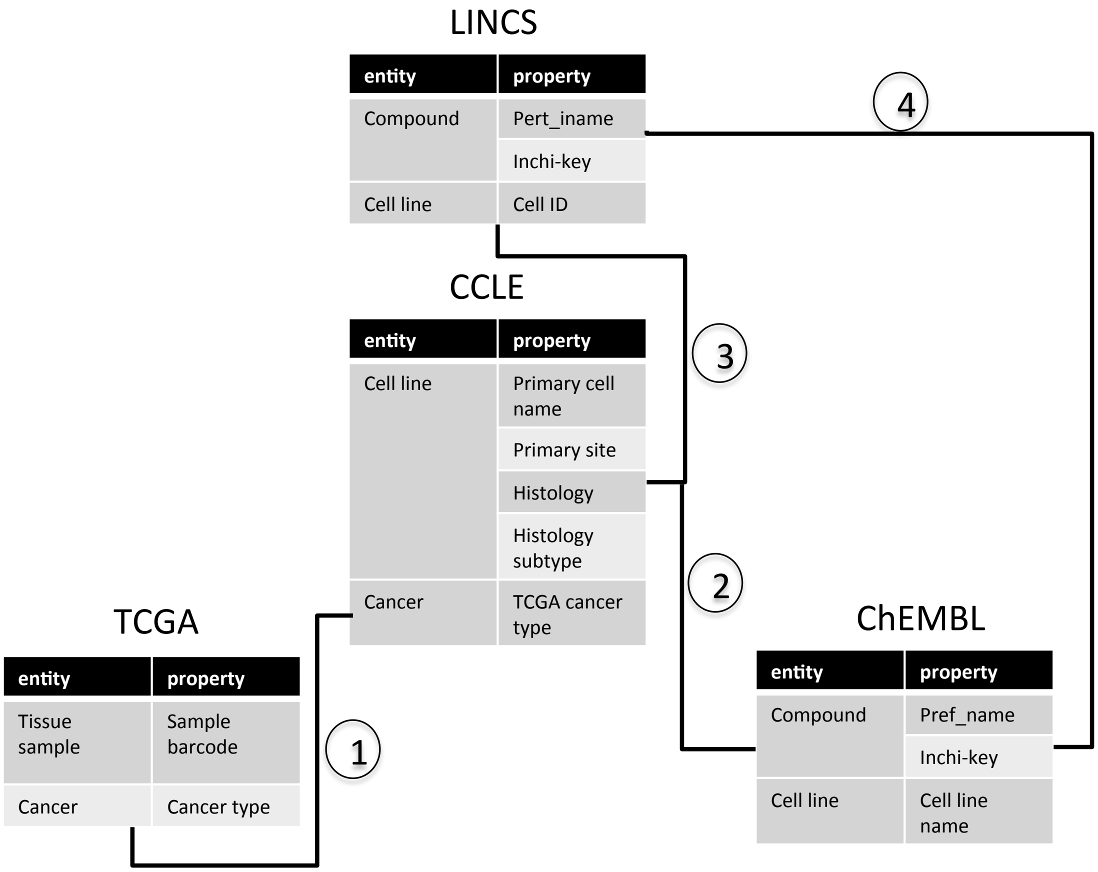

Supplementary Figure 9: Schema of data harmonization. Detailed description is in Supplementary Methods.

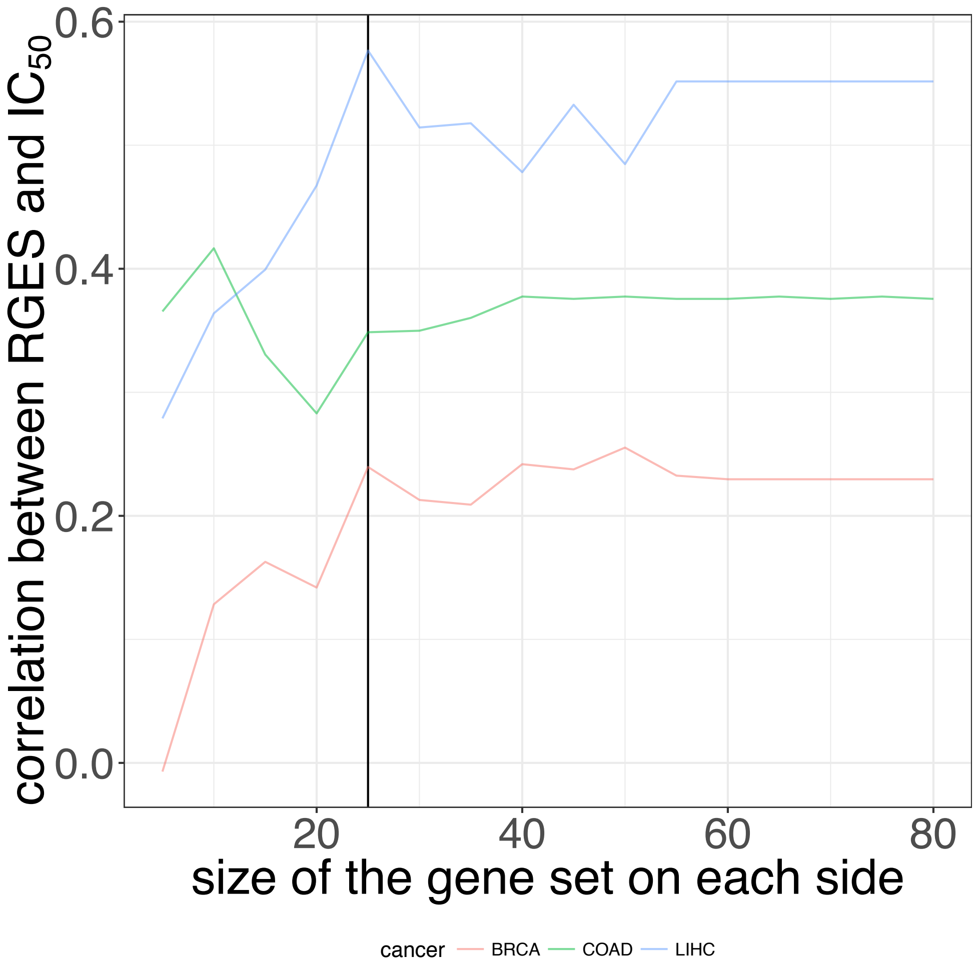

Supplementary Figure 10: Correlation between RGES and IC_{50} using different sizes of gene sets to build disease signatures. The plot shows that the correlation decreases when the size of gene set decreases, suggesting that drug efficacy is more difficult to predict for disease signatures with fewer differentially expressed genes.

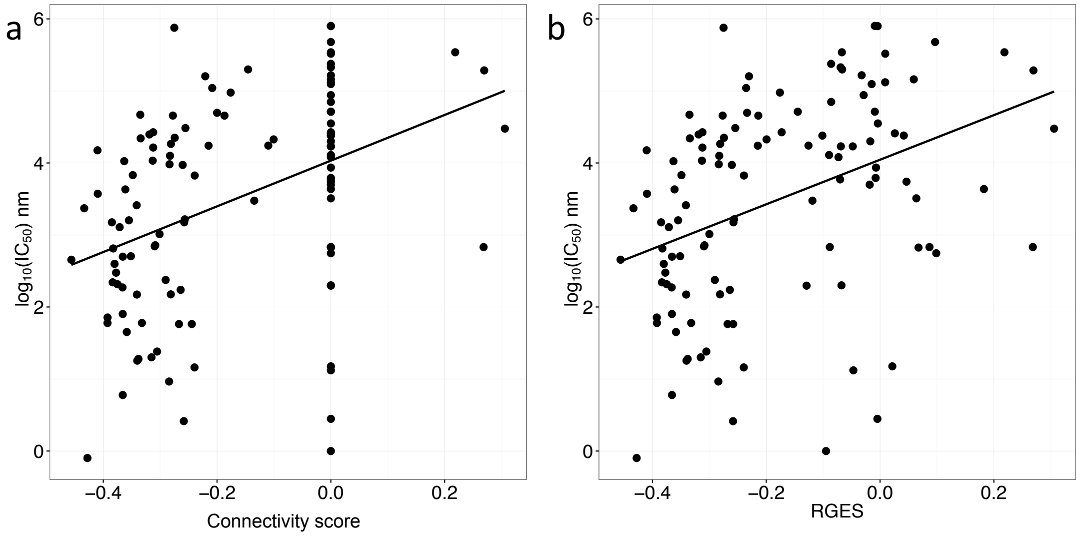

Supplementary Figure 11: Correlation between IC_{50} and (a) connectivity score (b) RGES in BRCA. The large number of connectivity scores enriched at 0 could affect the correlation. We only used BRCA as an example, but the bias exists in other cancers as well.

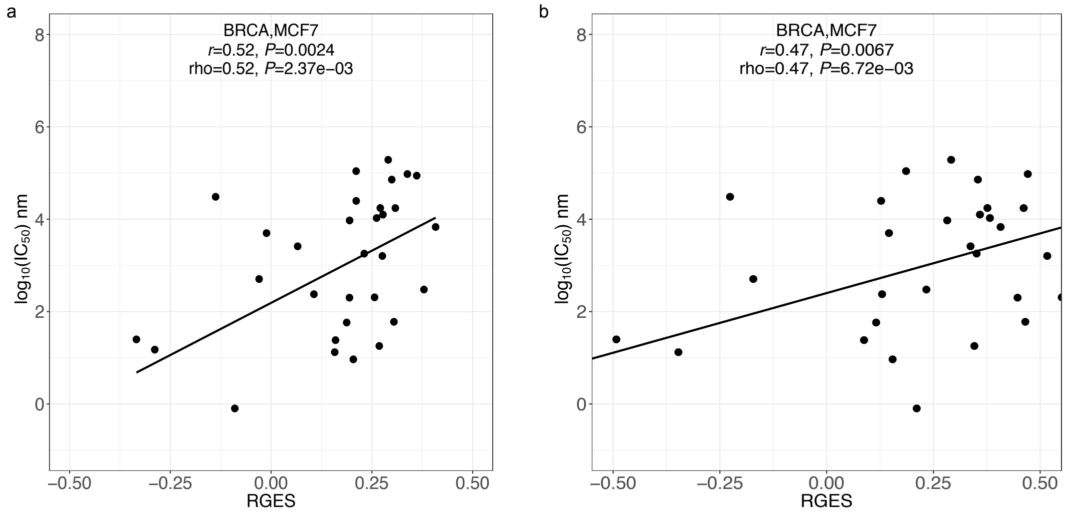

Supplementary Figure 12: Correlation between drug efficacy and RGES (a) using expression of the whole genome, (b) using the expression of landmark genes. Drug gene expression profiles were retrieved from CMap, which includes one breast cancer cell line

MCF7. Median was used to summarize multiple RGES. ANOVA and Spearman correlation were used to measure correlation between RGES and drug efficacy.

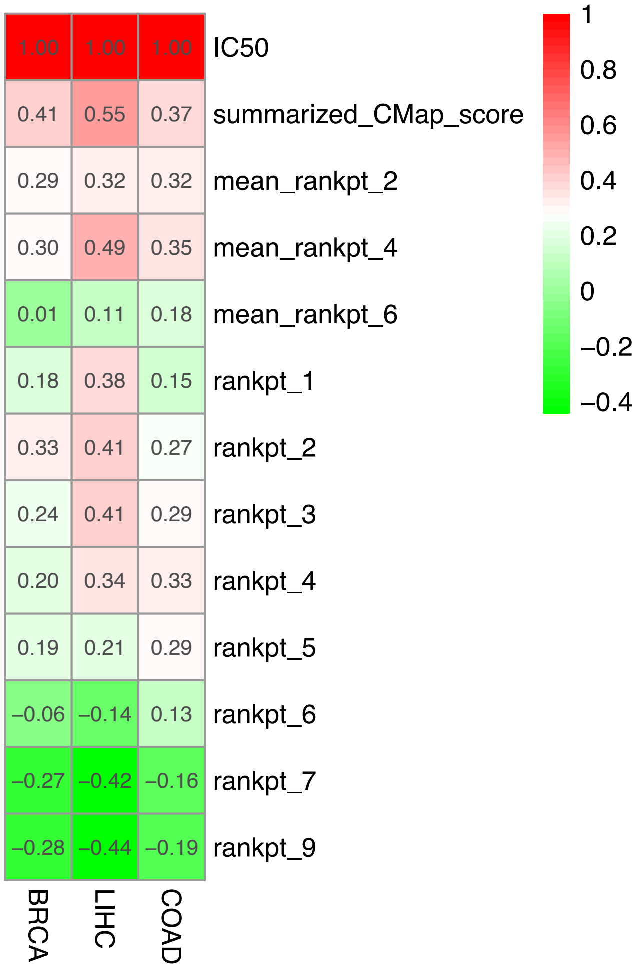

Supplementary Figure 13: Correlation between IC_{50} and summarization methods using the results generated from the LINCS cloud. Summarized_CMap_score represents the method we developed to summarize scores and the rest are the methods provided in the LINCS cloud. Different from existing methods, our method incorporated the confounding factors (cell line, treatment conditions) into our computation. The heat maps show that our method led to the best correlation with drug efficacy.

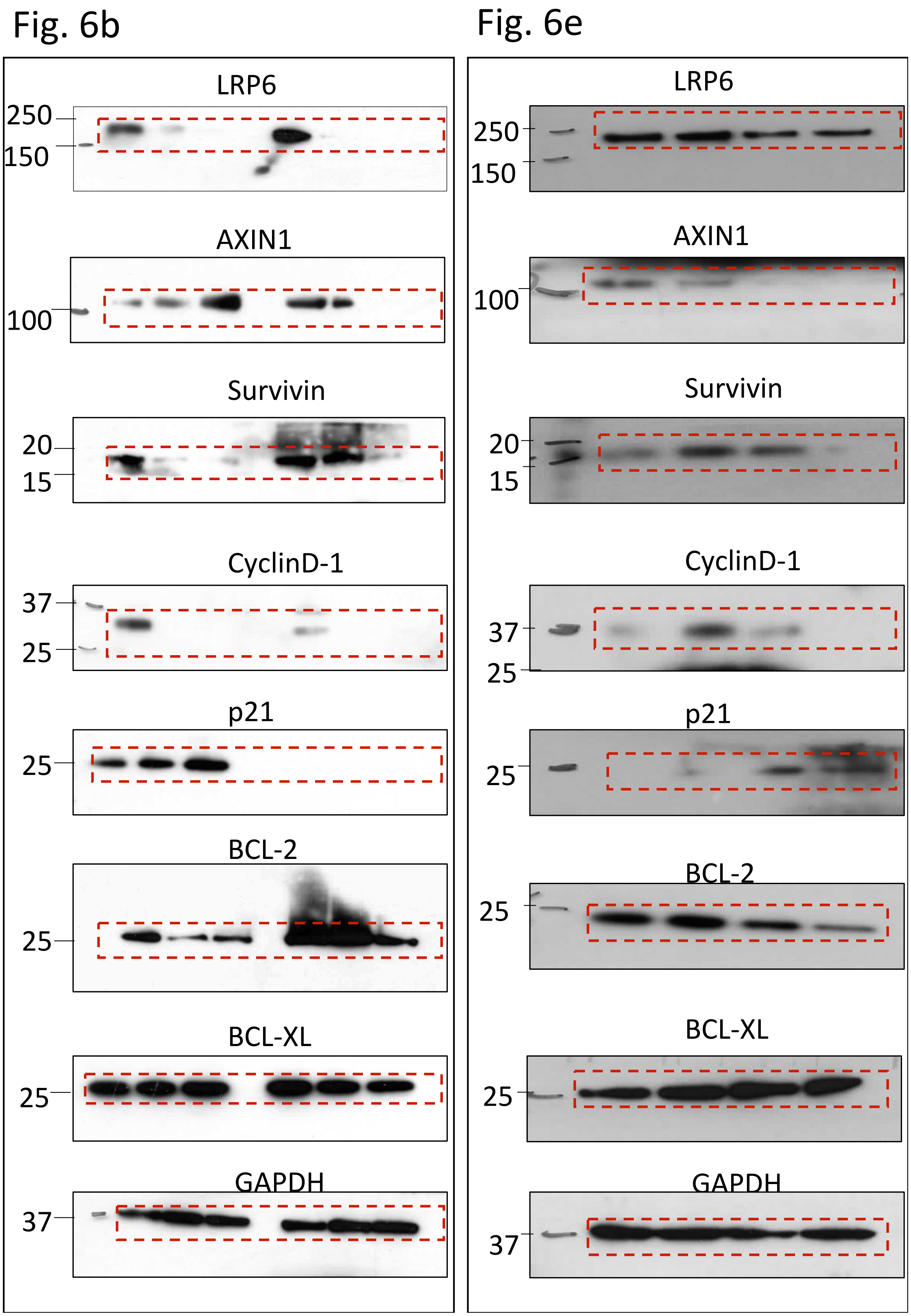

Supplementary Figure 14: Full versions of cropped western blots.
